# The association of interferon‐alpha with development of collateral circulation after artery occlusion

**DOI:** 10.1002/clc.23734

**Published:** 2021-10-02

**Authors:** Zhenhua Xing, Xiaopu Wang, Junyu Pei, Zhaowei Zhu, Shi Tai, Xinqun Hu

**Affiliations:** ^1^ Department of Emergency Medicine, The Second Xiangya Hospital Central South University Changsha China; ^2^ Department of Cardiovascular Medicine, The Second Xiangya Hospital Central South University Changsha China

**Keywords:** collateral circulation, coronary chronic total occlusion, IFN‐alpha

## Abstract

**Background:**

Previous studies have demonstrated that interferon (IFN) signaling is enhanced in patients with poor collateral circulation (CC). However, the role and mechanisms of IFN‐alpha in the development of CC remain unknown.

**Methods:**

We studied the serum levels of IFN‐alpha and coronary CC in a case–control study using logistics regression, including 114 coronary chronic total occlusion (CTO) patients with good coronary CC and 94 CTO patients with poor coronary CC. Restricted cubic splines was used to flexibly model the association of the levels of IFN‐alpha with the incidence of good CC perfusion restoration after systemic treatment with IFN‐alpha was assessed in a mice hind‐limb ischemia model.

**Results:**

Compared with the first IFN‐alpha tertile, the risk of poor CC was higher in the third IFN‐alpha tertile (OR: 4.79, 95% CI: 2.22–10.4, *p* < .001). A cubic spline‐smoothing curve showed that the risk of poor CC increased with increasing levels of serum IFN‐alpha. IFN‐alpha inhibited the development of CC in a hindlimb ischemia model. Arterioles of CC in the IFN‐alpha group were smaller in diameter than in the control group.

**Conclusion:**

Patients with CTO and with poor CC have higher serum levels of IFN‐alpha than CTO patients with good CC. IFN‐alpha might impair the development of CC after artery occlusion.

## 1 BACKGROUND

Development of collateral circulation (CC), also termed as arteriogenesis, is a natural life‐preservation mechanism occurring in patients with coronary chronic total occlusion (CTO). Well‐developed CC preserves cardiac function, thus reducing cardiac mortality after coronary occlusion.^1^, ^2^ Patients with CTO show a great deal of heterogeneity in their arteriogenic responses to artery occlusion. This is affected by multiple factors, including inflammatory factors.^3^ The interaction between inflammation and arteriogenesis may be worthy of study, but data on the correlation between arteriogenesis and inflammation are limited. A previous study has found that interferon (IFN) signaling in monocytes is enhanced and stimulated by lipopolysaccharide among patients with impaired coronary CC.^4^ Furthermore, blocking the receptor of IFN have been found to be helpful in alleviating ischemia in mice.^5^ However, whether patients with poor CC have higher levels of serum IFN or whether treatment with IFN inhibits the development of arteriogenesis remains unknown. IFN‐alpha is widely used in treating hepatitis B and C, as well as various cancers. IFN‐alpha might affect the development of CC in patients with CTO. In this study, we aimed to investigate whether patients with poor CC have higher levels of IFN‐alpha, and if so, whether IFN‐alpha inhibits the development of CC.

## 2 MATERIALS AND METHODS

### 2.1 Study population

This study was conducted in accordance with the Declaration of Helsinki and with written informed consent of each participant. This study was approved by the medical ethics committee of Second Xiangya Hospital of Central South University. Patients undergoing coronary angiography at our catheter laboratory between January 2018 and June 2019 and with at least one major coronary artery total occlusion were enrolled in our study. The exclusion criteria were as follows: (1) patients with ST‐segment elevated myocardial infarction/non‐ST‐segment elevated myocardial infarction, (2) patients with severe cardiac dysfunction/cardiac shock (HYHA IV or Killip IV), (3) patients with coronary artery bypass grafting, (4) patients with malignant tumor, (5) patients with inflammatory or infectious diseases, and (6) patients with severe hepatic (Child class C) and renal dysfunction (glomerular filtration rate <15 mL/min/1.73 m^2^ or needing hemodialysis). Pei JY and Wang XP were blinded to the characteristics of the included patients. They reviewed the angiography results and classified the extent of coronary circulation by the Rentrop classification from 0 to 3 as follows: 0 = none, 1 = filling of side branches of the artery dilated via collateral channels without visualization of the epicardial segment, 2 = partial filling of the epicardial segment via collateral channels, and 3 = complete filling of the epicardial segment of the artery being dilated via collateral channels.^6^ Rentrop 0–1 were classified as poor CC development; Rentrop 2–3 were classified as good CC development. Venous blood samples were collected immediately before coronary angiography. Serum was separated at 4°C, and the levels of IFN‐alpha were measured by ELISA (BMS216, Invitrogen).

### 2.2 Ischemic hind‐limb model and laser speckle imaging

The animal protocol was approved by the animal ethics committee of Second Xiangya Hospital of Central South University. All animal works were performed in Second Xiangya Hospital of Central South University. Male C57/BL mice were anesthetized with 3% isoflurane. The left femoral artery was ligated and excised as described previously.^7^ The right leg underwent sham operation and was used as control. The mice were randomly divided into two groups: an INF‐alpha group with intraperitoneal injection of 2000 IU IFN‐alpha, and a control group with intraperitoneal injection of the same amount of saline. The IFN‐alpha concentration used is comparable to dosages used in patients. Hind‐limb prefusion was measured by laser speckle imaging under temperature‐controlled conditions, before and after left hind‐limb ligation, as well as at 3 days, 1 week, and 3 weeks following ligation. Color‐coded images of the paws representing the flux value were used to calculate the ratio of the tissue perfusion of the occluded (left) to the Sham‐operated (Sham) paw. The right hind‐limb was used as reference. All mice were put to death by cervical dislocation.

### 2.3 Histological analyses

Adductor muscles from bilateral hind limbs were harvested at 3 weeks after surgery and underwent immediate tissue fixation overnight. For mouse arteriole density identification, the adductor muscles were stained with alpha‐SMA monoclonal antibody at 3 weeks after ligation.

### 2.4 Statistical analyses

We presented baseline characteristics of patients as frequencies and percentages for categorical variables and as means and standard deviations or interquartile range for continuous variables, depending on whether data distribution was normal (assessed by normal Q‐Q plots). We compared categorical variables using chi‐square analysis, and continuous variables were compared by analysis of variance test or Mann–Whitney *U* test, according to distribution type. We evaluated the relationship between serum levels of IFN‐alpha and CC development with the use of the levels of IFN‐alpha as both a continuous and a categorical variable. We constructed logistic regression model to calculate the odds ratio (OR) among tertiles. We used the first tertile as reference and used the following three models: model 1: unadjusted; model 2: adjusted age, sex, hypertension, hyperlipidemia, smoking; model 3: adjusted age, sex, hypertension, hyperlipidemia, smoking, uric acid, CRP. We also used a two‐piecewise linear regression model to examine the threshold effect of the levels of IFN‐alpha on the risk of poor CC according to the smoothing plot. The threshold value was determined using a trial method which was to move the trial turning point along the predefined interval and picked up the one which gave maximum model likelihood. A log likelihood ratio test was conducted comparing one‐line linear regression model with two‐piecewise linear model. We further used restricted cubic splines with five knots at the 5th, 35th, 50th, 65th, and 95th centiles to flexibly model the association of the levels of IFN‐alpha with the incidence of good CC adjusted for model 3 where the threshold value served as the reference.

We also did several sensitivity analyses by excluding patients with age <55 years or >70 years or by using the quantiles for the levels of serum IFN‐alpha. We performed all of the analyses using R version 3.4.3 (R Foundation for Statistical Computing, Vienna, Austria).

## 3 RESULTS

Between January 2018 and June 2019, 208 patients with CTO were included in our study as required by the inclusion and exclusion criteria (Figure S1). The baseline characteristics of the included patients are presented in Table 1. No significant difference between groups was observed except ejection fraction and the levels of serum IFN‐alpha. In patients with poor CC, serum INF‐alpha levels were significantly higher (87.7 ± 42.0 vs. 59.8 ± 18.5, *p* < .001), and ejection fraction was significantly lower (51.2 ± 10.5 vs. 59.8 ± 18.5, *p* = .04) than in patients with good CC.

**TABLE 1:** Baseline characteristics of included patients

	Poor CC (*n* = 114)	Good CC (*n* = 94)	*p*‐value
Age (years)	61.9 ± 10.7	59.8 ± 10.8	.168
Male (*n*, %)	72 (76.6)	89 (78.1)	.464
Smoking (*n*, %)	49 (52.1)	62 (54.4)	.426
Diabetes (*n*, %)	37 (39.4)	40 (35.1)	.311
Hypertension (*n*, %)	42 (56.0)	58 (67.4)	.146
Hyperlipemia (*n*, %)	27 (28.7)	42 (36.8)	.138
WBC (10^9^/L)	7.25 ± 2.14	7.19 ± 1.94	.612
PLT (10^9^/L)	231 ± 64.1	214 ± 69.5	.542
Uric acid (μmol/L)	368 ± 105	351 ± 88.7	.191
Creatinine	94.7 ± 74.7	83.3 ± 44.1	.172
Total cholesterol (mmol/L)	4.00 ± 0.981	3.98 ± 1.06	.958
LDL (mmol/L ± SD)	2.48 ± 0.868	2.46 ± 0.983	.179
Triglyceride (mmol/L)	1.77 ± 1.02	1.86 ± 1.25	.598
CRP (mg/L ± SD)	10.5 ± 17.8	11.9 ± 23.7	.629
Ejection fraction (%)	51.2 ± 10.5	54.1 ± 9.47	.040
Occluded vessels (*n*, %)			
RCA	43 (45.7)	58 (50.9)	.275
LAD	24 (32.0)	29 (33.7)	.497
LCX	30 (31.9)	32 (28.1)	.326
Drugs			
Aspirin (%)	109 (95.6)	89 (94.7)	.990
Clopidogrel (%)	83 (72.8)	64 (74.4)	.554
Β‐blocker (%)	70 (61.2)	55 (58.5)	.778
Statin (%)	106 (93.0)	87 (92.6)	.880
IFN‐alpha (pg/ml)	87.7 ± 42.0	59.8 ± 18.5	<.001

Abbreviation: CC, collateral circulation; CRP, C‐reactive protein; LAD, left anterior descending; LCX, left circumflex; LDL, low density lipoprotein; PLT, platelet count; RCA, right coronary artery; WBC, white blood cells.

**FIGURE 1:**
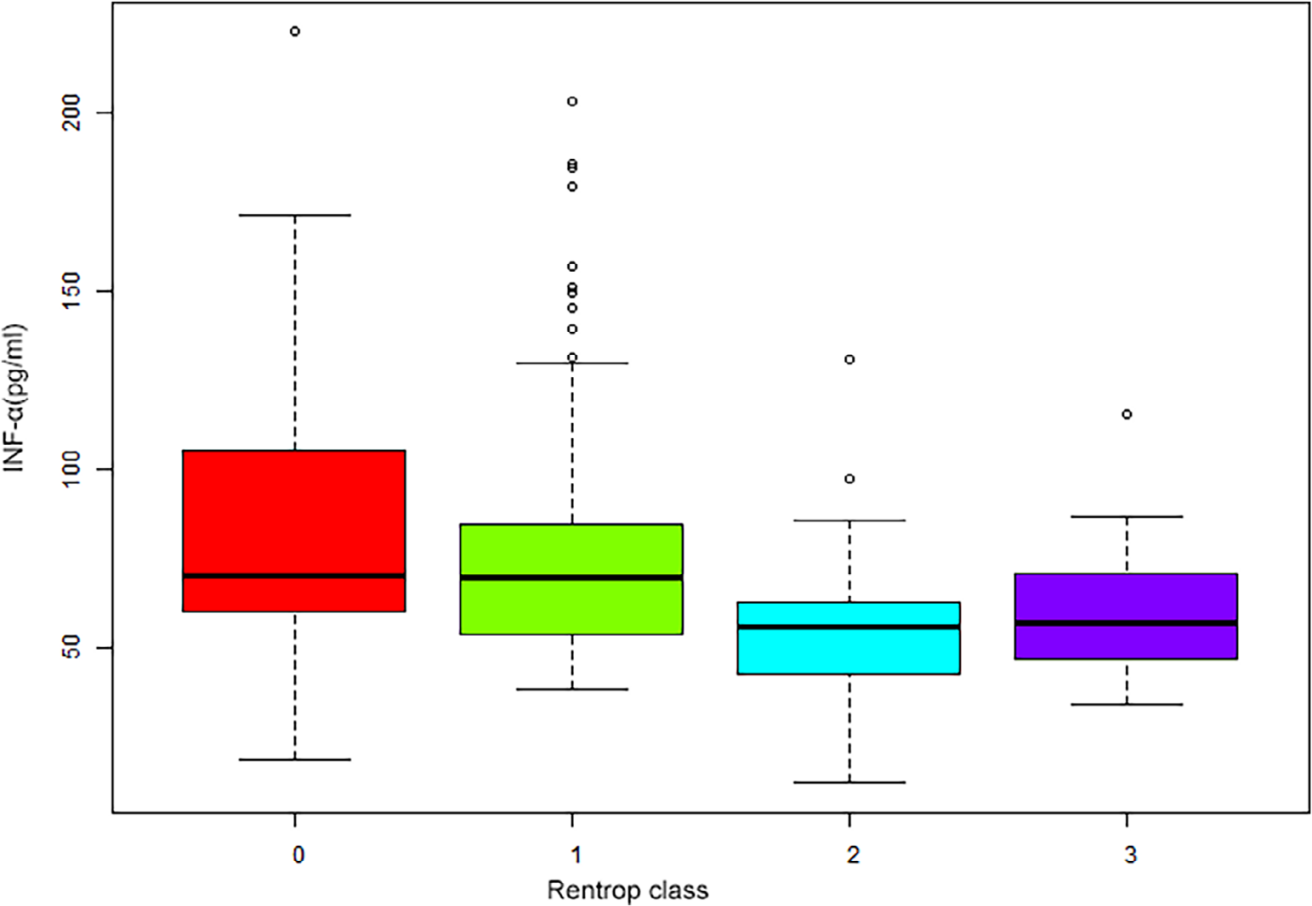
Correlation between the serum levels of IFN‐alpha and Rentrop scores. The levels in patients with Rentrop scores 0 and 1, or Rentrop 2 and 3, were not significantly different. The serum levels of IFN‐alpha in CTO patients with Rentrop scores of 2 and 3 were significantly different from those with Rentrop scores of 0 and 1

**FIGURE 2:**
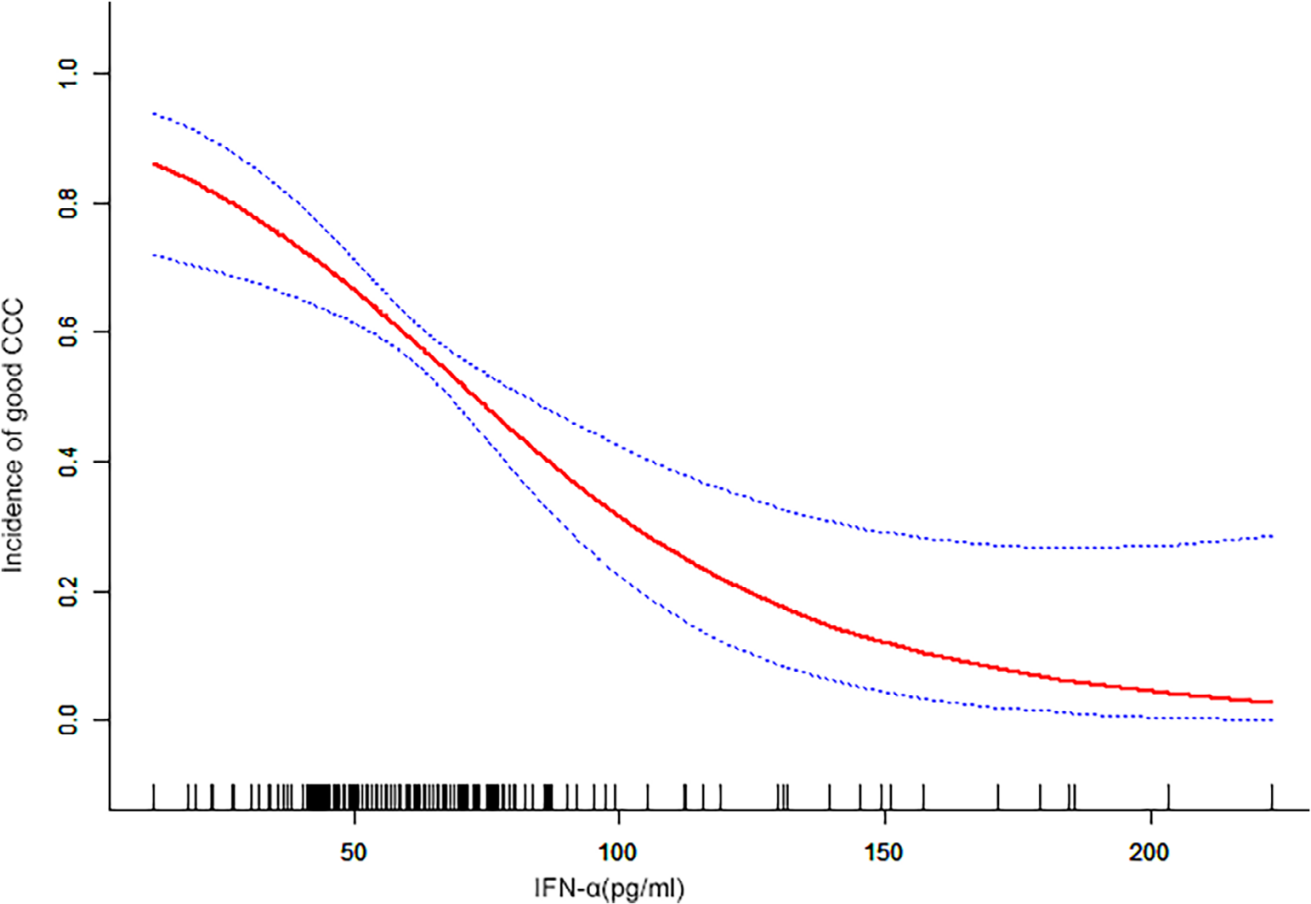
Relationship between the serum levels of IFN‐alpha and CC. A nonlinear relationship was observed between the serum levels of IFN‐alpha and CC after adjusting for model 3. Threshold effect analysis found that when serum levels of IFN‐alpha were larger than 112 pg/mL, the risks of poor CC did not keep on increasing

ANOVA showed that the serum levels of IFN‐alpha were significantly associated with CC, as did the Rentrop score (Figure 1). In a multivariate regression analysis, the risk of poor CC was increased with increasing IFN‐alpha tertile. Compared with the first tertile, the risk of poor CC was 3.67 (95% CI: 1.18–7.43) for model 1, 4.37 (95% CI: 2.06–9.26) for model 2, and 4.79 (95% CI: 2.22–10.4) for model 3 (Table 2). After adjusting for model 3, the cubic spline‐smoothing curve shows that the risk of poor CC increased with increasing serum IFN‐alpha levels (Figure 2). The incidence of poor CC increased with the serum levels of IFN‐alpha when the serum levels of IFN‐alpha were less than 112 pg/mL (OR: 1.0348, 95% CI: 1.0195–1.0577, *p* < .001, Table 3). When the levels of IFN‐alpha were larger than 112 pg/mL, the risk of poor CC did not increase with increasing levels of IFN‐alpha (OR: 1.0036, 95% CI: 0.9748–1.033, *p* = .807, Table 3). Our results remained robust in sensitivity analyses when we excluded patients aged >70 years or <55 years, as well as when using the fourth tertiles for the levels of serum IFN‐alpha (Tables S1–S3).

**TABLE 2:** OR (95% CI) of poor CC according to tertiles of the levels of serum IFN‐alpha

	Model 1		Model 2		Model 3	
	Odd ratio (95%CI)	*p*‐value	Odd ratio (95%CI)	*p*‐value	Odd ratio (95%CI)	*p*‐value
Tertile 1	Reference		Reference		Reference	
Tertile 2	1.11 (0.555–2.21)	.772	1.18 (0.582–2.41)	.641	1.26 (0.611–2.59)	.611
Tertile 3	3.67 (1.18–7.43)	<.001	4.37 (2.06–9.26)	<.001	4.79 (2.22–10.4)	<.001
*p* for trend	<.001		<.001		<.001	

*Note*: Model 1: unadjusted; model 2: adjusted age, sex, hypertension, hyperlipidemia, smoking; model 3: adjusted age, sex, hypertension, hyperlipidemia, smoking, uric acid, CRP.

**TABLE 3:** Threshold effect analysis of the serum levels of IFN‐alpha on poor CC

The serum levels IFN‐alpha	Odd ratio (95%CI)	*p*‐value
<112	1.0384 (1.0195, 1.0577)	<.001
≥112	1.0036 (0.9748, 1.0333)	.807

*Note*: When the levels of IFN‐alpha larger than 112 pg/mL, the incidence of poor CC did not increase with the increase of the levels of IFN‐alpha. Adjusted for model 3.

Abbreviation: CC, coronary collateral circulation.

**FIGURE 3:**
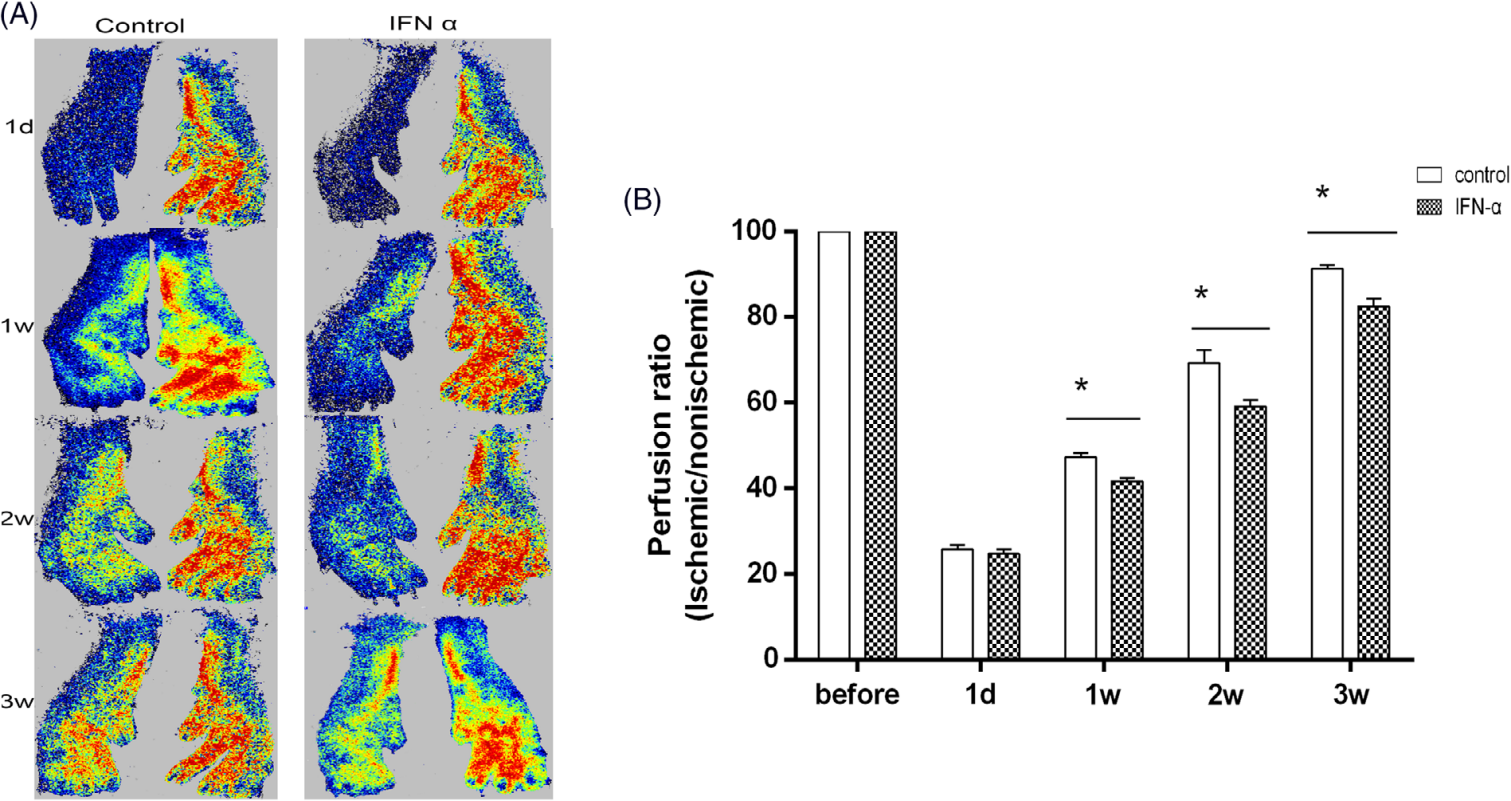
INF‐alpha impairs the development of CC in a murine hindlimb ischemic model. (A) Representative laser speckle perfusion images. (B) Quantification of laser speckle perfusion (ischemic/nonischemic) in control and IFN‐alpha treated mice over time

**FIGURE 4:**
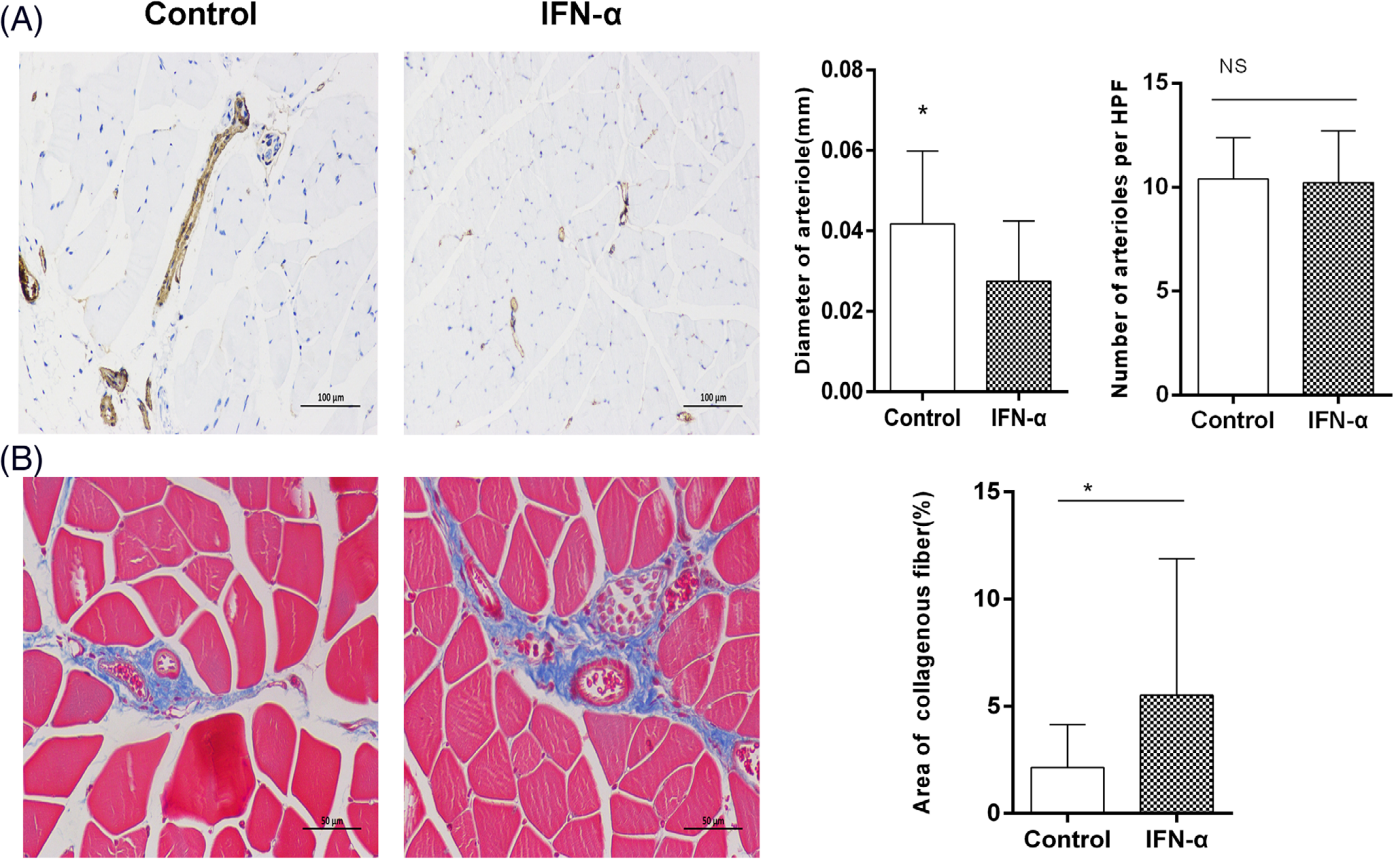
Immunohistochemistry of SMA in cross‐sections of the adductor muscles or Masson staining of gastrocnemius muscle collected from the control and IFN‐alpha group 3 weeks after femoral artery ligation. (A) Representative immunohistochemistry and quantification of CC numbers and diameter of adductor muscles. (B) Representative Masson staining and quantification of interstitial fibrosis of gastrocnemius muscle

To assess the in vivo effect of IFN‐alpha on CC, we examined whether intraperitoneal injection of IFN‐alpha at 2000 IU/d inhibited blood perfusion recovery in a mouse hind‐limb. Laser speckle imaging showed that blood flow recovery was significantly impaired in mice treated with IFN‐alpha compared with control mice at 1 week (41.7% ± 2.66% vs. 47.3% ± 2.16%, *p* = 0.020), at 14 days (59.8% ± 3.31% vs. 66.5% ± 3.21%, *p* = .005) and at 21 days (59.8% ± 3.31% vs. 66.5% ± 3.21%, *p* = .005, Figure 3A,B). Although the numbers of arterioles are comparable between groups, the diameter of the arterioles in the mice treated with IFN‐alpha was smaller compared with the control group (Figure 4A). In order to study local ischemia, we examined the morphology in the gastrocnemius muscle by HE staining and Masson staining. Although we did not find obvious morphological changes by HE analysis (Figure S2), the area of interstitial fibrosis evaluated by Masson staining in the mice treated with IFN‐alpha was larger than in the control group (Figure 4B).

## 4 DISCUSSION

In this study, we first found that CTO patients with poor CC have higher serum levels of IFN‐alpha. We found that IFN‐alpha inhibits the development of CC after artery occlusion.

The development of CC has a close relationship with multiple factors affecting coronary occlusion, such as diabetes mellitus, exercise, age, and other factors.^8^, ^9^, ^10^ Recent studies have found that inflammatory factors are involved in the progress of CC. Fan et al. found that C‐reactive protein was positively associated with poor CC in patients with coronary artery disease.^11^ Rakhit et al.^12^ found that tumor necrosis factor alpha (TNF‐alpha) was inversely correlated with collateral flow index (CFI), whereas IL‐6 was positively correlated with CFI. However, these case–control studies did not determine the cause and effect relationship between inflammatory factors and the development of CC. We further investigated this cause and effect relationship in animal experiments and found that IFN‐alpha inhibited the development of CC after hind‐limb ischemia.

The development of CC depends on local activation of endothelial cells, the eNOS signaling pathway.^13^ Previous study has found IFN‐alpha inhabited the expression and phosphorylation of eNOS in endothelial cells.^14^ Epstein found that aging caused collateral rarefaction by inhibiting eNOS; overexpression of eNOS restored normal CC.^15^ Furthermore, exercise promoted CC by promoting the expression and phosphorylation of eNOS. Therefore, eNOS is an essential factor for the development of CC. IFN‐alpha inhibits the expression and phosphorylation of eNOS, which may impair the development of CC. The positive remodeling of an arteriole into an artery up to 12–20 times its original size requires the proliferation and migration of endothelial cells and VSMCs.^16^, ^17^ Relevant study also found inflammatory factors, such as hs‐CRP, TNF‐alpha IFN‐alpha, inhibits the proliferation and migration of endothelial cells.^11^, ^12^ This may be another reason for patients with high serum levels of IFN‐alpha have impaired CC.

### 4.1 Limitation

First, the main limitation of present study is the small number of patients with CTO. However, the sensitivity analysis showed the robustness of our results. Second, our study did not include all inflammatory and angiogenic factors, they may have a role in the development of CC. Third, we evaluated the extent of CC by Rentrop classification instead of CFI. Although collateral is more accurate than Rentrop classification, it is an invasive procedure needing pressure wire. Rentrop classification is more often used in routine clinical practice. Fourth, we evaluated the development of CC in a model of hind‐limb ischemia rather than heart ischemia. However, both development of lower limb CC and coronary CC share common cellular and molecular mechanism: arteriogenesis. Ischemic hind‐limb model is an ideal model for arteriogenesis including coronary circulation.^4^, ^5^ Ischemic hind‐limb model mimics aspects of human occlusive artery disease to investigate vascular regeneration and to test therapeutical approaches in a reproducible manner.

## 5 CONCLUSION

CTO patients with poor CC have higher serum levels of IFN‐alpha than CTO patients with good CC. IFN‐alpha might impair the development of CC after artery occlusion.

## CONFLICTS OF INTEREST

The authors declare that they have no potential conflicts of interests.

## AUTHOR CONTRIBUTIONS

Xinqun Hu and Zhenhua Xing designed the study and provided methodological expertise. Zhenhua Xing drafted the manuscript. Zhenhua Xing performed the case–control study. Xiaopu Wang, Junyu Pei, Zhaowei Zhu, Shi Tai performed the animal experiments. All authors have read, provided critical feedback on, and approved the final manuscript.

## Supporting information


**Supplementary Table S1.** OR (95% CI) of poor CC according to fourths of the levels of serum IFN‐alpha
**Supplementary Table S2.** OR (95% CI) of poor CC by excluding patients with age >70 years
**Supplementary Table S3.** OR (95% CI) of poor CC by excluding patients with age <55 years
**Supplementary Figure S1.** Chart of inclusion and exclusion of present study
**Supplementary Figure S2.** HE staining of the left and right sides of gastrocnemius muscle.no obvious morphological changes were found

## Data Availability

All data generated or analyzed during this study are included in this published article. The datasets used and/or analyzed during the current study are available from the corresponding author on reasonable request.
