# Supplementary material for: The association of interferon‐alpha with development of collateral circulation after artery occlusion
Source: Clin Cardiol. 2021 Oct 2;44(11):1621–7. doi: 10.1002/clc.23734 (PMC8571556; doi:10.1002/clc.23734)
Supplement: Supplementary file 1 — Supplementary Table S1. OR (95% CI) of poor CC according to fourths of the levels of serum IFN‐alpha Supplementary Table S2. OR (95% CI) of poor CC by excluding patients with age >70 years Supplementary Table S3. OR (95% CI) of poor CC by excluding patients with age <55 years Supplementary Figure S1. Chart of inclusion and exclusion of present study Supplementary Figure S2. HE staining of the left and right sides of gastrocnemius muscle.no obvious morphological changes were found [file CLC-44-1621-s001.docx]

**The role and mechanisms of IFN-α in the development of collateral circulation after artery occlusion**

Running title: IFN-α and collateral circulation

Zhenhua Xing^2^, Junyu Pei^1,^ Xiaopu Wang^1^, Zhaowei Zhu^1^, Shi Tai^1^, Xinqun Hu^1^*

1 Department of Cardiovascular Medicine, The Second Xiangya Hospital，Central South University，Changsha，Hunan 410011，China

2 Department of Emergency Medicine, Second Xiangya Hospital, Central South University, Changsha, 410011, China.

*Corresponding author: Xinqun Hu, M.D

Email: huxinqun@csu.edu.cn

Phone number: +861584714930

**Supplementary Table1: OR(95% CI) of poor CC according to fourths of the levels of serum IFN-α**

|  | Model 1 | | | Model 2 | | | Model 3 | | |
| --- | --- | --- | --- | --- | --- | --- | --- | --- | --- |
| Groups | Odd ratio(95%CI) | P-value | | Odd ratio(95%CI) | P-value | | Odd ratio(95%CI) | P-value | |
| 1 | Reference | |  | Reference | |  | Reference | |  |
| 2 | 0.70（0.31-1.61） | | 0.40 | 0.67（0.29-1.6） | | 0.356 | 0.73（0.31-1.7） | | 0.611 |
| 3 | 1.7（0.77-3.6） | | 0.20 | 1.9（0.87-4.4） | | 0.106 | 2.1（0.91-4.7） | | <0.001 |
| 4 | 3.5 (1.5-7.8) | | 0.003 | 4.4 (1.9-10.3) | | 0.01 | 5.0 (2.1-12.2) | | <0.001 |
| P for trend | 0.001 | |  | <0.001 | |  | <0.001 | |  |

Model 1: unadjusted; Model 2: adjusted age, sex, hypertension, hyperlipidemia, smoking; Model 3 adjusted age, sex, hypertension, hyperlipidemia, smoking, uric acid, CRP.

**Supplementary Table 2 OR(95% CI) of poor CC by excluding patients with age ＞70 years**

|  | Model 1 | | | Model 2 | | | Model 3 | | |
| --- | --- | --- | --- | --- | --- | --- | --- | --- | --- |
|  | Odd ratio(95%CI) | P-value | | Odd ratio(95%CI) | P-value | | Odd ratio(95%CI) | P-value | |
| Tertile 1 | Reference | |  | Reference | |  | Reference | |  |
| Tertile 2 | 1.1（0.49-2.3） | | 0.888 | 1.2（0.52-2.6） | | 0.727 | 1.2（0.53-2.7） | | 0.66 |
| Tertile 3 | 3.1（1.4-6.6） | | 0.004 | 3.6（1.6-8.4） | | <0.002 | 3.9（1.7-9.1） | | 0.02 |
| P for trend | 0.005 | |  | 0.003 | |  | 0.003 | |  |

Model 1: unadjusted; Model 2: adjusted age, sex, hypertension, hyperlipidemia, smoking; Model 3 adjusted age, sex, hypertension, hyperlipidemia, smoking, uric acid, CRP.

**Supplementary Table 3: OR(95% CI) of poor CC by excluding patients with age ＜55 years**

|  | Model 1 | | Model 2 | | | Model 3 | | |
| --- | --- | --- | --- | --- | --- | --- | --- | --- |
|  | Odd ratio(95%CI) | P-value | Odd ratio(95%CI) | P-value | | Odd ratio(95%CI) | P-value | |
| Tertile 1 | Reference |  | Reference | |  | Reference | |  |
| Tertile 2 | 1.3（0.57-2.8） | 0.568 | 1.4（0.58-3.2） | | 0.477 | 1.5（0.63-3.6） | | 0.357 |
| Tertile 3 | 5.2（2.1-13） | <0.001 | 5.5（2.1-14） | | <0.001 | 6.6（2.4-18） | | <0.001 |
| P for trend | 0.001 |  | 0.001 | |  | 0.001 | |  |

Model 1: unadjusted; Model 2: adjusted age, sex, hypertension, hyperlipidemia, smoking; Model 3 adjusted age, sex, hypertension, hyperlipidemia, smoking, uric acid, CRP.


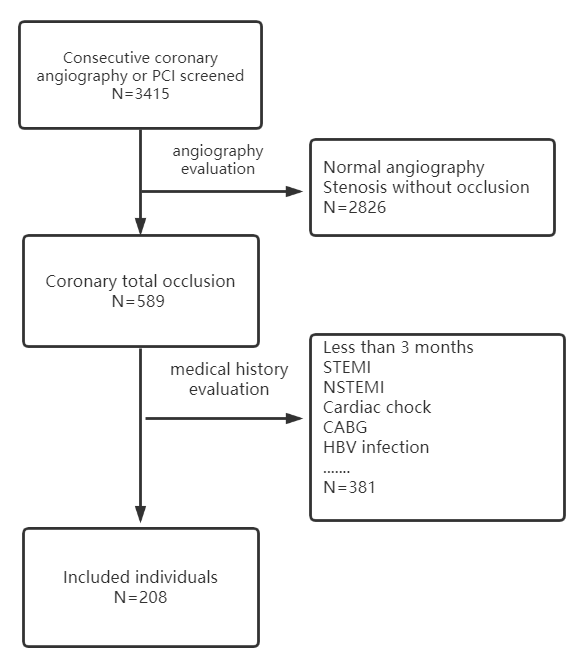


**Supplementary Figure 1: Chart of inclusion and exclusion of present study**


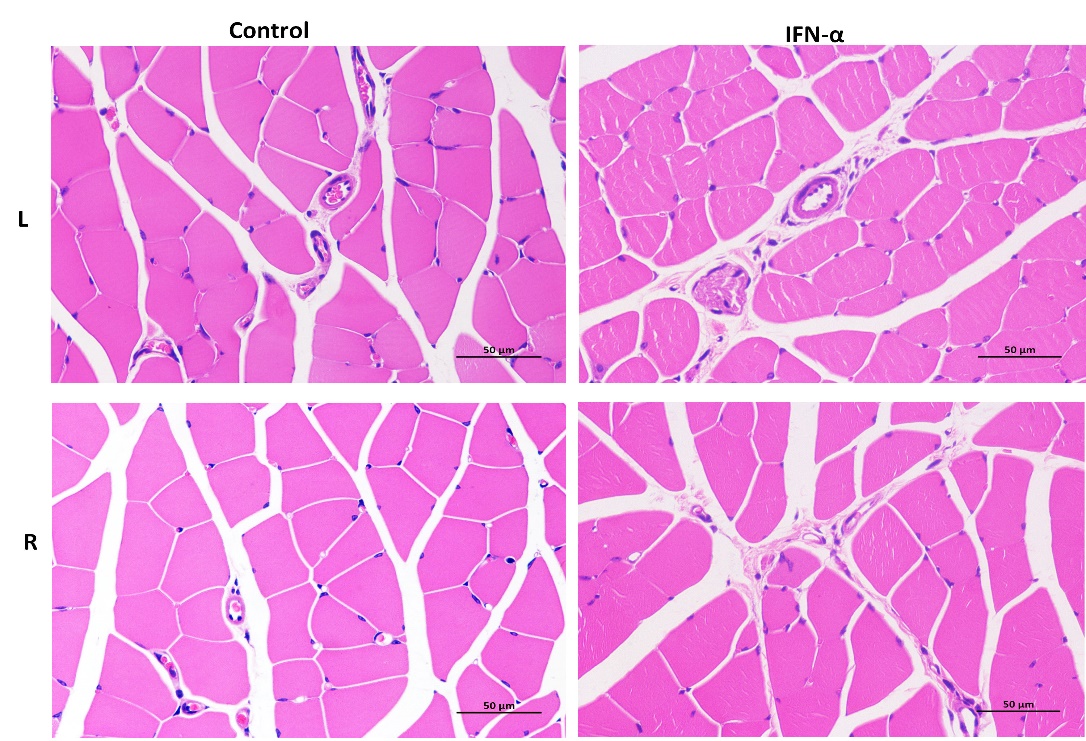


**Supplementary Figure 2: HE staining of the left and right sides of gastrocnemius muscle.no obvious morphological changes were found.**
